# Low‐dose aspirin use and colorectal cancer survival in 32,195 patients—A national cohort study

**DOI:** 10.1002/cam4.4859

**Published:** 2022-06-19

**Authors:** Mehrnoosh Shahrivar, Caroline E. Weibull, Karin Ekström Smedby, Bengt Glimelius, Ingvar Syk, Peter Matthiessen, Caroline Nordenvall, Anna Martling

**Affiliations:** ^1^ Department of Molecular Medicine and Surgery Karolinska Institutet Stockholm Sweden; ^2^ Department of Medicine Solna, Clinical Epidemiology Division Karolinska Institutet Stockholm Sweden; ^3^ Department of Immunology, Genetics and Pathology Uppsala University Uppsala Sweden; ^4^ Department of Surgery Skåne University Hospital Malmö Sweden; ^5^ Department of Surgery, Faculty of Medicine and Health Örebro University Örebro Sweden; ^6^ Department of Pelvic Cancer, GI oncology and colorectal surgery unit Karolinska University Hospital Stockholm Sweden

**Keywords:** aspirin, colorectal cancer, pharmacoepidemiology, survival

## Abstract

**Background:**

Results from previous studies indicate that use of aspirin may improve colorectal cancer (CRC) survival. The aim of this study was to assess whether use of aspirin influences overall survival or CRC‐specific survival in an unselected cohort of patients diagnosed with CRC.

**Methods:**

The study was performed using the Colorectal Cancer Data Base Sweden (CRCBaSe), a mega‐linkage originating from the Swedish Colorectal Cancer Register, with additional linkages to other national health care registers. All patients diagnosed with primary CRC stage I–III treated with curative surgery, aged 18–85 years at diagnosis, from 2007 through 2016 were identified. Information on low‐dose aspirin use was extracted from the Swedish Prescribed Drug Register. Exposure was defined as dispensed prescription for at least 6 months. Aspirin exposure was analyzed at the time of surgery (yes/no) and as a time‐varying exposure during follow‐up. Follow‐up was restricted to a maximum 6 years, to model 5‐year survival. Cox regression models were fitted to estimate hazard ratios (HRs) with 95% confidence intervals (CIs). Adjustments were performed for sex, age, year of diagnosis, Charlson comorbidity index, hypertension, and ASA score as potential confounders.

**Results:**

A total of 32,195 patients diagnosed with CRC were included. 6764 (21%) were exposed to aspirin at the time of CRC surgery. The median time of follow‐up was 4.2 years. Aspirin use at the time of surgery was not associated with all‐cause (adjusted HR = 1.03, 95% CI: 0.97–1.08) nor CRC‐specific mortality (adjusted HR = 0.99, 95% CI: 0.91–1.07). Aspirin use during follow‐up was associated with increased all‐cause (adjusted HR = 1.09, 95% CI: 1.04–1.15) but not CRC‐specific mortality (adjusted HR = 0.98, 95% CI: 0.91–1.06). A CRC‐specific effect associated with aspirin was noted from approximately 3 years following surgery.

**Conclusions:**

In this large nation‐wide cohort study there was no convincing association between aspirin use after CRC and OS or CRC‐specific survival.

## Graphical abstract

It is well established that regular use of aspirin can reduce the relative risk of developing colorectal cancer. However, the impact of aspirin on survival after colorectal cancer diagnosis is not well understood. Studies have shown conflicting results. In this population‐based cohort study, the largest one to date, of all patients diagnosed with colorectal cancer in 2007‐2016, we found that aspirin was not associated with neither overall, colorectal cancer‐specific or disease‐free survival.

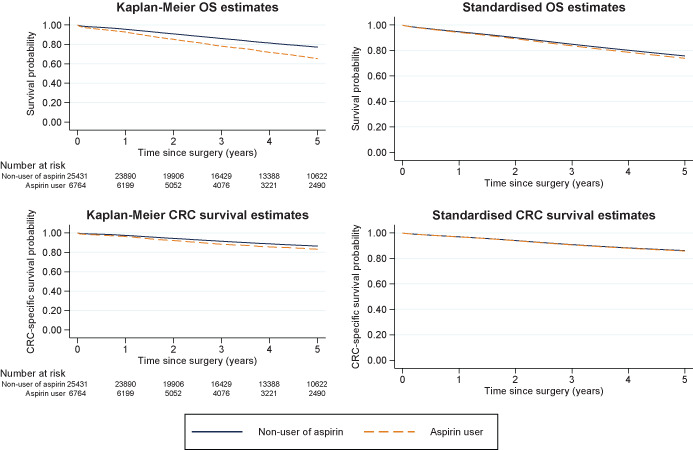

## 1 INTRODUCTION

Colorectal cancer (CRC) is the second leading cause of cancer mortality, representing 9% of all cancer deaths worldwide.^1^ The global burden of CRC is expected to increase with 60% by 2030,^2^ and more efficient adjuvant therapies are needed to improve survival of CRC.

A large number of studies indicate that regular use of aspirin may reduce the risk of developing CRC by 16%–44%.^3^, ^4^, ^5^, ^6^, ^7^ These results have led the US Preventive Service Task Force in 2016 to recommend aspirin use as primary prevention of CRC in adults aged 50–59 years who have a 10% or greater 10‐year cardiovascular disease risk, are not at increased risk for bleeding and have a life expectancy of at least 10 years.^8^

Studies of the association between aspirin use and CRC prognosis have presented varying results. While some have shown no association,^9^, ^10^, ^11^ others have reported a reduction in both all‐cause and cancer‐specific mortality,^12^, ^13^, ^14^, ^15^, ^16^ or in all‐cause mortality only.^17^, ^18^ Several of these studies have demonstrated limitations such as small sample size, self‐reported exposure, short duration of aspirin intake, and lack of adjustments for confounders. However, more recently a randomized controlled trial of adults aged 70 years or older, on the contrary, showed an increased risk of cancer‐related mortality.^19^ Additional evidence is therefore needed to determine whether aspirin use is associated with improved overall survival (OS) and/or CRC‐specific survival in an unselected cohort of patients diagnosed with CRC.

The present study included all patients with stage I–III CRC undergoing surgery with curative intent in Sweden during a 10‐year period, with detailed information on dispensed prescription and clinical variables retrieved from national registers, to address the question of a possible association between aspirin use and outcome among CRC‐patients. We hypothesized that post‐diagnosis aspirin use is associated with an improved OS and CRC‐specific survival.

## 2 MATERIALS AND METHODS

### 2.1 Data sources

The study was performed within Colorectal Cancer Data Base Sweden (CRCBaSe Sweden). CRCBaSe contains all patients with a CRC diagnosis registered in the Swedish Colorectal Cancer Register (SCRCR), their relatives and matched comparators. Using the personal identification number unique to all Swedish residents, CRCBaSe was created by linking patient data from the SCRCR to several nationwide health and demographic registers. Relevant for the study at hand are the Swedish Cancer Register, the Cause of Death Register, the Swedish Prescribed Drug Register, the National Patient Register, and the Register of Total Population.

The SCRCR is a nationwide population‐based quality‐of‐care register with data on patients diagnosed with rectal (since 1995) and colon (since 2007) cancer. It contains detailed information on tumor characteristics, treatment, and recurrence. The completeness of the SCRCR is 98.5%,^20^ and was here used to identify all CRC cases in the cohort and collect data on sex, year of diagnosis, age at diagnosis, American Society of Anesthesiologists (ASA) score, tumor location, tumor stage, tumor differentiation grade, surgery, and recurrence.

The Swedish Cancer Register (SCR) was established in 1958 and contains all newly diagnosed primary cancers. Reporting to the register is mandatory by law, and the completeness is >96%.^21^ The SCR was used to identify previous cancer diagnosis. The Cause of Death Register contains information on date of death and underlying and contributing causes of death (coded according to the international classification of disease [ICD] system) on all deceased Swedish residents since 1961.^22^ It has a completeness of >99% and high cause‐of‐death accuracy for malignant neoplasms, 90%–98%.^23^

The Swedish Prescribed Drug Register was established in 2005 and is a nationwide register on all dispensed medications, apart from over‐the‐counter (OTC) medications and drugs used in hospitals and nursing homes.^24^ All drugs are classified according to the Anatomic Therapeutic Chemical (ATC) code. It contains data on dispensed items, date of prescription and dispensing, dispensed amount, and dosage.

The National Patient Register has nationwide coverage of all inpatient care since 1987 and outpatient specialist visits since 2001. Primary care is not covered. Diagnoses are coded according to the ICD system.^25^ Together with the SCR, the register was used to assess the comorbidity burden for CRC patients. The Charlson Comorbidity Index (CCI) was calculated based on diagnoses, excluding colorectal cancer C18‐C19, within five (for the National Patient Register) and 10 (for the SCR) years of CRC diagnosis.^26^ ICD codes used to calculate CCI index can be found in Table S1. The presence of hypertension, not included in CCI, was identified using ICD‐10 code I10.

The Register of Total Population contains demographic information including residence, sex, civil status, and immigrations/emigrations, for the entire population of Sweden. The register was used for administrative censoring (due to migrations) purposes and to match each CRC‐patient in the cohort with population comparators.

### 2.2 Study population

The study population was identified through CRCBaSe, and included all patients diagnosed with a primary stage I–III CRC between 2007 and 2016 at ages 18–85 years, who had undergone surgical resection with curative intent (*n* = 32,200). Five additional patients were excluded due to inconsistent death dates, yielding a final study population of 21,266 colon cancer patients and 10,929 rectal cancer patients (*n* = 32,195).

### 2.3 Aspirin exposure definition

Information on low‐dose aspirin use was extracted from the Swedish Prescribed Drug Register (ATC codes B01AC06). Low‐dose aspirin is only available by prescription in Sweden and in two dosages, 75 and 160 mg and cannot be purchased OTC. High‐dose aspirin (ATC codes N02BA01, N02BA51, and N02AJ09) constituted 1.6% of all aspirin prescriptions in this study population (own data) and were not included in the study. Exposure to aspirin was defined as a dispensed prescription covering a treatment for a period of 180 days or more. In event of multiple dispense, gaps sizes of twice the duration of the most recent dispense were allowed. Patients with a dispense that fulfilled these criteria within the year before CRC surgery or during follow‐up (regardless of if they had more dispenses or discontinued aspirin use) were considered exposed from the first dispense date in that episode. Patients unexposed at the time of surgery were those never having used aspirin, having <180 days of use, or having a 180+ days use but not within the year prior to CRC surgery. These patients could later become exposed if they had a dispense fulfilling the criteria above during follow‐up.

### 2.4 Outcome definition

The two primary outcomes of interest were all‐cause and CRC‐specific mortality, where the latter was defined as deaths with CRC registered as the underlying cause of death (ICD‐10 C18.0‐C18.9, C19, C20). As a secondary outcome, relapse‐free survival (RFS; recurrence/metastasis/all‐cause death) was investigated. Date of recurrence/metastasis was extracted from SCRCR. All endpoints were defined according to Punt et al.^27^

### 2.5 Comparators

To assess the association between aspirin use and all‐cause mortality in a cancer‐free cohort of individuals similar to the CRC‐patients, each patient was matched (on year of birth and sex) to six population comparators free of CRC at the time of patient diagnosis. A total of 193,165 comparators were included in this sensitivity analysis.

### 2.6 Statistical analysis

Data was analyzed using survival analysis, with time since surgery as the underlying time scale throughout. The start of follow‐up was the date of CRC surgery. Patients were followed up for a maximum of 6 years, until date of death, emigration, or end of study period (31 December 2017), whichever occurred first. Follow‐up was restricted to the first 6, rather than 5 years after diagnosis to improve stability of the modeled 5‐year overall and CRC‐specific survival estimates.^28^ Cox proportional hazards models were used to estimate hazard ratios (HRs) and 95% confidence intervals (CIs). For demographical and clinical characteristics, univariable models were fitted. To assess the association between aspirin use and all‐cause/CRC‐specific mortality, a series of analyses were performed. Firstly, patients exposed to aspirin at the time of CRC surgery were contrasted to the non‐users. Secondly, two approaches to analyze aspirin as a time‐varying exposure were taken. In the first one, aspirin exposure was treated as a binary variable and patients were considered exposed from the dispense date when accumulating 180 days of aspirin use within the same episode. In case this occurred before the start of follow‐up, patients were classified as exposed from the start of follow‐up. With this approach, no separation between those exposed from start and those becoming exposed was made. Secondly, a differentiation between patients on aspirin already at the time of surgery and those starting after CRC surgery (still using the date of accumulating 180 days of use within the same episode) was made to account for potential indication bias in these two groups, meaning that three contrasts are presented (aspirin use at CRC surgery vs. start of aspirin use after CRC surgery vs. non‐aspirin use, with the last group containing unexposed follow‐up time as well).

When analyzing RFS, only the contrast between aspirin exposure at surgery was investigated.

Adjustments for potential confounders were performed using three sets of models: 1, no adjustments; 2, adjustment for age at CRC surgery, sex, year of diagnosis; 3, adjustment for the same variables plus comorbidity (coded as CCI and hypertension) and ASA score. The assumption of proportional hazards (PH) was formally evaluated using Schoenfeld residuals.

Survival proportions (OS and CRC‐specific survival) were calculated by aspirin exposure at CRC surgery, both using the Kaplan–Meier method and standardized over the observed age, sex, year of diagnosis, CCI, and hypertension distribution, using a flexible parametric survival model with five degrees of freedom for the baseline hazard.^29^, ^30^ A similar (but additionally adjusted for ASA score) model for all‐cause and CRC‐specific mortality was also fitted where the effect of aspirin use at the time of CRC surgery was allowed to vary over follow‐up (i.e., non‐PH). For the time‐varying effect, 3 degrees of freedom were used.

A number of sensitivity analyses were performed. First, we adjusted for stage as a potential mediator. Secondly, analyses were carried out in which the material was stratified by variables such as age, sex, tumor location, and disease stage. Thirdly, sensitivity analysis using the complete study period, instead of 6 years restriction to measure 5‐year survival, was conducted. Finally, to investigate how well indication for aspirin use was captured and adjusted for, a sensitivity analysis was performed where OS‐ and CRC‐specific survival among aspirin exposed patients were compared to matched population comparators.

All statistical analyses were conducted in STATA v16 software (StataCorp). For the flexible parametric survival model, we used the Stata package stpm2, version 1.7.5. To predict standardized survival, we used package standsurv, version 0.6.^30^ Ethical approval was acquired from the Regional Ethical Board, Stockholm, Sweden (Dnr: 2014/71–31, 2018/328–32 and 2021–00342).

## 3 RESULTS

### 3.1 Baseline characteristics

A total of 32,195 CRC patients met the inclusion criteria and were followed‐up after the date of surgery for a median time of 4.2 years (interquartile range 2.2–6.0). One‐fifth of the cohort was exposed to aspirin at surgery (6764 patients). Patient characteristics by aspirin exposure are outlined in Table 1. The overall mean age at diagnosis was 69.5 years, with aspirin users having a higher mean age than non‐users (74.4 vs. 68.3 years). There was a male predominance in users (61.6% vs. 51.4%) and these patients also had more comorbidities (60.9% had CCI ≥ 1 vs. 31.8% in non‐users). Colon cancer was more common among aspirin users (70.0% vs. 65.0%), and aspirin users had overall a lower stage CRC, stage I (25.9% vs. 23.8%) and stage III cancer (35.3% vs. 37.8%).

**TABLE 1:** Frequencies and proportions of baseline clinical characteristics among 32,195 Swedish patients diagnosed with colorectal cancer, stratified by aspirin exposure at the time of surgery

	Aspirin user	Non‐user	Total	HR^b^ (95% CI)
Overall, *n* (row%)	6764 (21.0)	25,431 (79.0)	32,195 (100)	—
Dead^a^ *n* (col%)				
All‐cause deaths	2127 (31.4)	5188 (20.4)	7315 (22.7)	
CRC‐specific deaths	926 (13.7)	2867 (11.3)	3700 (11.5)	
Relapse^a^ *n* (col%)				
Yes	933 (13.8)	3651 (14.4)	4584 (14.2)	
No	1191 (17.6)	21,780 (85.6)	27,611 (85.8)	
Year of diagnosis *n* (col%)				
2007–2011	3238 (47.9)	11,685 (45.9)	14,923 (46.4)	1.00
2012–2016	3526 (52.1)	13,746 (54.1)	17,272 (53.7)	0.91 (0.86–0.95)
Age at diagnosis *n* (col%)				
18–49	17 (0.3)	1586 (6.2)	1603 (5.0)	0.68 (0.58–0.80)
50–59	216 (3.2)	3245 (12.8)	3461 (10.8)	0.70 (0.63–0.79)
60–69	1395 (20.6)	7739 (30.4)	9134 (28.4)	1.00
70–79	3233 (47.8)	9070 (35.7)	12,303 (38.2)	1.81 (1.70–1.93)
80–85	1903 (28.1)	3791 (14.9)	5694 (17.7)	3.27 (3.06–3.50)
Mean age (SD)	74.4 (7.3)	68.3 (10.8)	69.5 (10.5)	—
Sex *n* (col%)				
Male	4168 (61.6)	13,067 (51.4)	17,235 (53.5)	1.00
Female	2596 (38.4)	12,364 (48.6)	14,960 (46.5)	0.80 (0.77–0.84)
Tumor location *n* (col%)				
Colon, right	2730 (40.4)	9186 (36.1)	11,916 (37.0)	1.00
Colon, left	2006 (29.7)	7337 (28.9)	9343 (29.0)	0.80 (0.76–0.85)
Colon, UNS	2 (0.03)	5 (0.02)	7 (0.02)	—
Rectum	2026 (30.0)	8903 (35.01)	10,929 (34.0)	0.83 (0.79–0.88)
Disease stage *n* (col%)				
Stage I	1749 (25.9)	6055 (23.8)	7804 (24.2)	1.00
Stage II	2631 (38.9)	9767 (38.4)	12,398 (38.5)	1.35 (1.26–1.44)
Stage III	2384 (35.3)	9609 (37.8)	11,993 (37.3)	2.26 (2.12–2.42)
Tumor differentiation grade *n* (col%)				
Low‐grade	5285 (78.1)	19,959 (78.5)	25,244 (78.4)	1.00
High‐grade	1240 (18.3)	4409 (17.3)	5649 (17.6)	1.53 (1.45–1.62)
Missing	239 (3.5)	1063 (4.2)	1302 (4.0)	—
Charlson comorbidity index (CCI) *n* (col%)				
0	2643 (39.1)	17,343 (68.2)	19,986 (62.1)	1.00
1	1507 (22.3)	2278 (9.0)	3785 (11.8)	1.78 (1.66–1.91)
≥2	2614 (38.7)	5810 (22.9)	8424 (26.2)	2.11 (2.01–2.22)
ASA score *n* (col%)				
Score 1–2	3310 (48.9)	19,260 (75.7)	22,570 (70.1)	1.00
Score 3–5	3238 (47.9)	5402 (21.2)	8640 (26.8)	2.67 (2.54–2.80)
Missing	216 (3.2)	769 (3.0)	985 (3.1)	—

*Note*: Due to rounding, all percentages do not add up to 100%.

Abbreviations: CI, confidence interval; col, column; CRC, colorectal cancer; HR, hazard ratio; *n*, number.

^a^ Restricted to the first 6 years after CRC‐surgery date.

^b^ All‐cause mortality for each variable estimated from univariable Cox regression models.

During follow up at a total of 11,015 patients died, 7315 deaths from any cause and 3700 were secondary to CRC. A total of 4584 relapses were recorded of which 76 were excluded in the analysis as data was missing on date.

### 3.2 Aspirin use and all‐cause mortality

**FIGURE 1:**
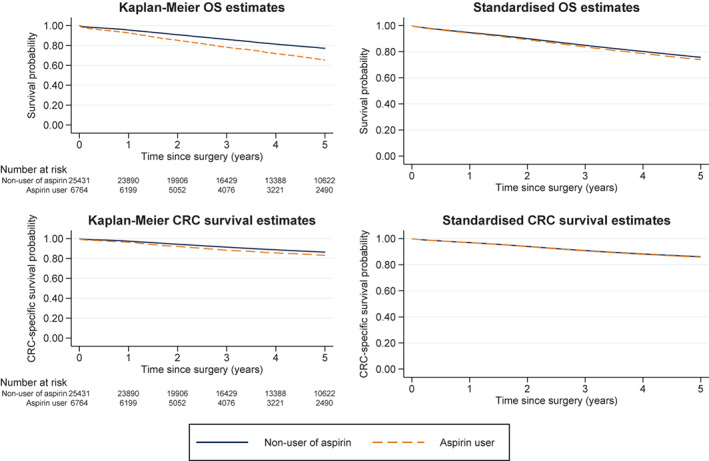
Overall survival (OS, top panel) and CRC‐specific survival (bottom panel) estimated with the Kaplan–Meier method (left panels) and standardized over age, sex, year of diagnosis, Charlson comorbidity index, and hypertension (right panels), by aspirin exposure at the time of CRC‐surgery

**FIGURE 2:**
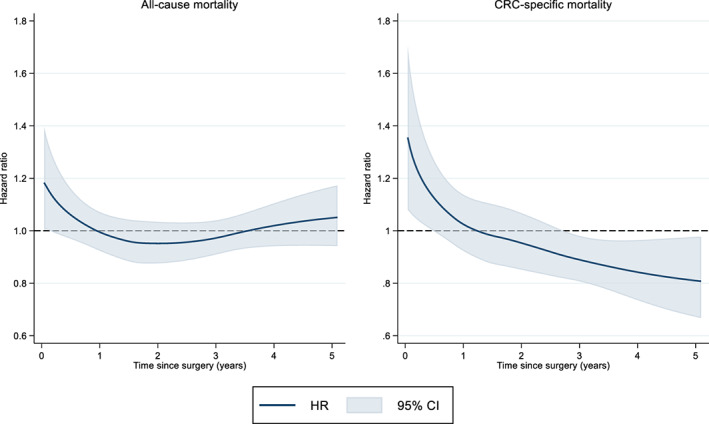
Time‐varying hazard ratios (HRs) with 95% confidence intervals (CIs) as measure of the association between aspirin use time of CRC surgery (non‐users as reference) and all‐cause (left panel) and colorectal cancer (CRC)‐specific mortality (right panel) in patients with colorectal cancer adjusted for age, sex, year of diagnosis, Charlson comorbidity index, hypertension, and ASA score

Aspirin use at surgery was associated with an increased mortality rate in univariable analyses, but no association was found in the multivariable models (HR = 1.03, 95% CI: 0.97–1.08) (Figure 1, Table 2). Allowing for non‐proportional hazards did not reveal any trend in aspirin effect over follow‐up (Figure 2). When analyzing aspirin use as a time‐varying exposure, a slight increase in all‐cause mortality was observed (adjusted HR = 1.09, 95% CI: 1.04–1.15), which was more prominent in those that had started to use aspirin after CRC surgery (adjusted HR = 1.36, 95% CI: 1.24–1.49) (Table 2).

**TABLE 2:** Hazard ratios (HRs) with 95% confidence intervals (CIs) comparing all‐cause (top) and CRC‐specific (bottom) mortality between aspirin users/non‐users in 32,195 CRC patients diagnosed between 2007–2016, aged 18–85 years

All‐cause mortality	HR^a^ (95% CI)	HR^b^ (95% CI)	HR^c^ (95% CI)
Aspirin use at CRC‐surgery date			
Non‐aspirin use	1.00 (ref)	1.00 (ref)	1.00 (ref)
Aspirin use^d^	1.64 (1.56–1.72)	1.24 (1.18–1.31)	1.03 (0.97–1.08)
Time‐varying exposure analyses			
Non‐aspirin use	1.00 (ref)	1.00 (ref)	1.00 (ref)
Aspirin use	1.75 (1.66–1.83)	1.33 (1.26–1.40)	1.09 (1.04–1.15)
Among aspirin‐users (pre‐ and post‐surgery)	1.72 (1.63–1.81)	1.29 (1.23–1.36)	1.04 (0.98–1.10)
Among non‐users (post‐surgery use only)	1.87 (1.71–2.05)	1.50 (1.36–1.64)	1.36 (1.24–1.49)
CRC‐specific mortality			
Aspirin use at CRC‐surgery date			
Non‐aspirin use	1.00 (ref)	1.00 (ref)	1.00 (ref)
Aspirin use^d^	1.28 (1.19–1.38)	1.08 (1.00–1.17)	0.99 (0.91–1.07)
Time‐varying exposure analyses			
Non‐aspirin use	1.00 (ref)	1.00 (ref)	1.00 (ref)
Aspirin use	1.29 (1.20–1.39)	1.09 (1.01–1.17)	0.98 (0.91–1.06)
Among aspirin‐users (pre‐ and post‐surgery)	1.30 (1.21–1.40)	1.09 (1.01–1.17)	0.98 (0.90–1.06)
Among non‐users (post‐surgery use only)	1.25 (1.08–1.46)	1.09 (0.93–1.26)	1.02 (0.87–1.19)
Relapse‐free survival (relapse or death)			
Aspirin use at CRC‐surgery date			
Non‐aspirin use	1.00 (ref)	1.00 (ref)	1.00 (ref)
Aspirin use^d^	1.42 (1.35–1.49)	1.17 (1.11–1.23)	1.01 (0.96–1.06)

*Note*: Time measured from date of CRC surgery and restricted to the first 6 years after surgery.

^a^ Estimated from an unadjusted Cox regression model.

^b^ Estimated from a model as in (a) also adjusting for age at diagnosis, sex, and year of diagnosis.

^c^ Estimated from a model as in (b) also adjusting for Charlson comorbidity index, hypertension, and ASA score.

^d^ Defined as having at least one prescription of aspirin equaling to or exceeding a total of 180 days dispense, within the year prior to CRC‐surgery date/index date.

### 3.3 Aspirin use and CRC‐specific mortality

Averaged across follow‐up, no association between aspirin use at surgery and CRC‐specific mortality was found in the multivariable models (HR = 0.99, 95% CI: 0.91–1.07) (Table 2). On the survival scale, the CRC‐specific survival (unadjusted and standardized) did not differ noticeably from OS (Figure 1). However, when allowing for non‐proportional hazards, a protective effect of aspirin was observed after approximately 3 years following surgery (Figure 2). In the analysis of aspirin use as a time‐varying exposure the PH results were the same (HR = 0.98, 95% CI: 0.91–1.06) (Table 2). Aspirin exposure at the time of surgery was not associated with improved RFS in the multivariable models (HR = 1.01, 95% CI: 0.96–1.06) (Table 2) and RFS (unadjusted and standardized) did not differ from OS and CRC‐specific survival (data not shown).

### 3.4 Sensitivity analyses

As an exploratory model, we adjusted for the potential mediator stage and the hazard ratio of all‐cause and CRC‐specific mortality remained unchanged (data not shown). When including an interaction with age, sex, tumor location, and disease stage (separately), no significant effects of aspirin use were seen within any of the different strata (Table S2). Whether patients were considered exposed at the dispense date or when they had accumulated 180‐days of use within the same episode, the results of all‐cause and CRC‐specific mortality were not noticeably different (data not shown). Sensitivity analysis using the complete study period did not change the results (Table S3).

Similar to the patients, aspirin users among comparators were older and had a higher CCI score (Table S4). Multivariable analyses showed inferior OS among comparator who were aspirin users (adjusted HR = 1.26, 95% CI: 1.23–1.30) compared to non‐users, despite adjustments for CCI and hypertension. The standardized OS indicated superior survival in comparators compared with CRC‐patients, indifferent of aspirin use (Figure S1).

## 4 DISCUSSION

In this nation‐wide study of 32,195 CRC patients, the largest to date, no convincing association between aspirin use and all‐cause or CRC‐specific mortality was found. Neither was aspirin use associated with RFS. However, the results showed a potential effect associated with aspirin from approximately 3 years following surgery.

Previous findings on pre‐diagnostic aspirin use and outcome following CRC have been inconsistent, some studies have observed a protective effect of pre‐diagnostic use^12^, ^16^, ^17^, ^31^ but most have not.^9^, ^10^, ^11^, ^32^, ^33^ Similarly, post‐diagnostic use has been associated with improved CRC survival in a few studies^12^, ^14^, ^16^, ^33^, ^34^, ^35^ whereas no association was reported by others.^9^, ^10^, ^11^ One randomized controlled trial has even shown an increased mortality in aspirin users, 70 years or older, following CRC diagnosis.^19^ On recurrence risk in CRC, very few studies have evaluated the impact of aspirin use. A single‐center study of 726 CRC patients showed a reduced risk of recurrence among aspirin users.^33^ Our study, the only population‐based cohort conducted in this field, did not show any association. These inconsistencies may have several explanations. In the present study, and in line with previous publications, aspirin users have more favorable stage at diagnosis.^11^, ^16^, ^31^, ^34^ Most studies have included stage I‐IV CRC patients^9^, ^10^, ^12^, ^14^, ^16^, ^17^, ^31^, ^34^, ^36^ and even though they have adjusted for stage, potential residual confounding may have affected the results. We tried to avoid this by restricting to patients with non‐metastasized CRC who had received curative treatment. Another possible explanation could be earlier detection in aspirin users due to regular health care visits or aspirin‐induced gastrointestinal bleeding from the tumor. Yet another explanation may be an anticancer effect of aspirin through its inhibition of cyclooxygenase (COX), a rate‐limiting enzyme in prostaglandin production.^37^ Studies in human cancer have shown increased COX‐2 expression in colon cancer,^37^ and in some studies it has been shown to impact the survival benefits associated with aspirin in CRC.^15^, ^38^ To achieve COX‐2 suppression, high aspirin dose is however required.^37^ Previous studies have described the important role of platelets in cancer growth, progression, and metastasis.^37^ The antiplatelet action of aspirin, through inhibition of COX‐1, could therefore be another explanation to the lower stage of CRC found among aspirin users.

Another aspect that differs among previous studies is the definition of exposure. Self‐reported use potentially introduces information bias whereas the present study is based on register information on dispensed drugs instead. Further, some studies required only aspirin prescription for 14 days or 1 month,^9^, ^12^, ^14^, ^34^, ^36^ possibly including short‐term users that may have temporarily used aspirin for pain‐relief in the exposed group. These differences in definition may partly explain the inconsistencies in the published data.

It is possible that there is an effect of aspirin on CRC prognosis in specific subgroups that we were unable to detect. The effect of aspirin use on survival after CRC diagnosis may differ according to tumor expression of COX‐2^15^, ^38^ and presence of PIKC3CA mutation,^39^ present in 11%–17% of CRC patients.^39^, ^40^, ^41^ These genetic and molecular factors of the tumors may contribute to the inconsistencies observed in the prognostic effect of aspirin on CRC survival. This information is not routinely analyzed in Sweden and given the large cohort of more than 32,000 patients with CRC, we were unable to obtain this information.

Furthermore, it is possible that both aspirin dose and duration is of importance. Results from two pooled American cohorts showed that a protective effect of aspirin use was only evident in younger (below 70 years) patients who had used aspirin for more than 5 years.^42^ As information on aspirin use was not available prior to July 2005, this longer exposure window was not possible to evaluate in the current study. Initially, those that were aspirin users at the time of surgery experienced an increased CRC‐specific mortality rate. However, 3 years *after* surgery the CRC‐specific mortality was lower in those that were aspirin users at the time of surgery compared to those who were not. Our result is in contrast with Bains et al, a population‐based cohort of 23,162 stage I‐IV CRC patients, where a protective effect was observed only *in* the first 2 years after diagnosis.^16^ Although interesting, we find no explanation of this discrepancy in the results, which also might be by chance. Further studies are needed to investigate these findings.

This study has some important strengths. First, it is the largest cohort investigating the connection between aspirin use and survival in CRC‐patients. Its nationwide coverage diminishes the risk of selection bias and makes the results generalizable to countries with a similar population. Second, data were retrieved from high‐quality and continuously updated registries with long and virtually complete follow‐up. Third, recall bias was avoided because data about aspirin use was based on *dispensed* medication.

There are a number of limitations to our study. First, despite excellent data on patient‐, CRC‐characteristics, and treatment from national registers, there is the potential for residual confounding. It was evident in the analyses of CRC‐free comparators that aspirin‐using comparators had inferior survival compared to non‐aspirin ‐using comparators, despite adjustments for comorbidity using CCI. Classification of comorbidities did not include information from primary care, and hence the complete comorbidity burden of patients might not have been fully captured. To reduce the risk of residual confounding of comorbidity, adjustments were made for CCI as well as ASA score. Second, OTC access of high‐dose aspirin is another possible source of bias as it is available both by prescription and as OTC. However, high‐dose OTC aspirin is available in small packages, is more expensive and not covered by the drug reimbursement system, unlike prescribed high‐dose aspirin, and therefore unlikely to affect the outcome. Also, it has been shown that valid treatment associations can be estimated using prescription databases when the overall prevalence of drug use is less than 35%, and the proportion of OTC drug exposure is as high as 80%.^43^ Although we had information for dispensed drugs, we lacked information on compliance. In addition, patients were considered exposed from the start of aspirin use, since there are no scientific criteria to define the most appropriate length of lag‐time for aspirin use.^44^, ^45^ Third, as mentioned previously, information on COX‐2 expression and PIK3CA mutation status, molecular and genetic biomarkers which could potentially impact the effect of aspirin on survival, were not available to us.^38^, ^39^, ^40^ Fourth, there is a possibility that some recurrences were not reported to the SCRCR. A recent report showed that 4% and 1.6% of recurrences were unreported before and after 5 years of follow‐up, respectively.^46^

In conclusion, in this large nation‐wide cohort study we did not find any evidence of a protective effect of aspirin after CRC on OS or CRC‐specific survival in a non‐selected patient population. There were signs of a potential CRC‐specific effect associated with aspirin from approximately 3 years following surgery that warrant further investigation. Randomized, placebo‐controlled biomarker‐based clinical trials are needed to evaluate the impact of adjuvant aspirin in CRC.

## AUTHOR CONTRIBUTIONS

Study conception and design: all authors. Collection and assembly of data: AM, CN, CW, and MS. Data analysis and interpretation: AM, CN, CW, and MS. Drafting or revising manuscript: all authors. Final approval for submission: all authors.

## FUNDING INFORMATION

The study was supported financially by the Swedish Research Council, the Swedish Cancer Society, the Stockholm Cancer Society, and supported by grants provided by Region Stockholm (ALF project).

## CONFLICT OF INTEREST

CEW is part of a research collaboration between Karolinska Institutet and Janssen Pharmaceutica NV for which Karolinska Institutet has received/receives grant support. The remaining authors have no disclosures or potential conflict of interest.

## Supporting information


Table S1–S4
Figure S1

## Data Availability

The data underlying this article can be shared upon request to the corresponding author.
