# Supplementary material for: Low‐dose aspirin use and colorectal cancer survival in 32,195 patients—A national cohort study
Source: Cancer Med. 2022 Jun 19;12(1):315–24. doi: 10.1002/cam4.4859 (PMC9844641; doi:10.1002/cam4.4859)
Supplement: Supplementary file 1 — Table S1–S4 Figure S1 [file CAM4-12-315-s001.docx]

**S TABLE 1** International classification of disease (ICD) codes used to calculate Charlson Comorbidity index.

| **Comorbidity** | **ICD-10 code** |
| --- | --- |
| Acute myocardial infarction | I21, I22, I25.2 |
| Congestive heart failure | I11.0, I13.0, I13.2, I25.5, I42.0, I42.6, I42.7, I42.8, I42.9, I43, I50 |
| Peripheral vascular disease | I70, I71, I73.1, I73.8, I73.9, I77.1, I79.0, I79.2, K55 |
| Cerebral vascular disease | G45, I60, I61, I62, I63, I64, I67, I69 |
| Chronic obstructive pulmonary disease | J43, J44 |
| Other chronic pulmonary disease | J41, J42, J45, J46, J47, J60, J61, J62, J63, J64, J65, J66, J67, J68, J69, J70 |
| Rheumatic disease | M05; M06, M12.3, M07.0–3, M08, M13, M30, M31.3-M31.6, M32, M33, M34, M35.0, M35.1, M35.3, M45-46 |
| Dementia | F00, F01, F02, F03, F05.1, G30, G31.1, G31.9 |
| Hemiplegia, tetraplegia | G11.4, G80, G81, G82, G83.0-G83.3, G83.8 |
| Diabetes | E10.0, E10.1, E11.0-E11.1, E12.0-E12.1, E13.0-E13.1, E14.0-E14.1 |
| Diabetes with end organ damage | E10.2, E10.3, E10.4, E10.5, E10.7, E11.2-E11.7, E12.2-E12.7, E13.2-E13.7, E14.2-E14.7 |
| Moderate or severe kidney disease | N03.2-N03.7, N05.2-N05.7, N11, N18, N19, N25.0, I12.0, I13.1, Q61.1-Q61.4, Z49, Z94.0, Z99.2 |
| Mild liver disease | B15-B19, K70.3, K73, K74.6, K70.3, K75.4 |
| Moderate or severe kidney disease | N03.2-N03.7, N05.2-N05.7, N11, N18, N19, N25.0, I12.0, I13.1, Q61.1-Q61.4, Z49, Z94.0, Z99.2 |
| Peptic ulcer disease | K25, K26, K27, K28 |
| Any malignancy, including leukemia and lymphoma | C00-C97*, not C87 |
| Metastatic cancer | C77, C78, C79, C80 |
| HIV/AIDS | B20, B21, B22, B23, B24, F02.4, O98.7, R75, Z11.4, Z21.9, Z71.1 |

* Excluding colorectal cancer C18-C20

**S TABLE 2** Hazard ratios (HRs) with 95% confidence intervals (CIs) of the association between aspirin use at time of CRC surgery and all-cause mortality (non-aspirin users is the reference group), stratified by age, sex, tumor location, and stage.

| **Effect modifier** |  | **HR** (95% CI)** | |
| --- | --- | --- | --- |
| **Age at diagnosis** | 18-49 | 1.00 | 0.69 (0.17-2.77) |
|  | 50-59 | 1.00 | 0.87 (0.60-1.26) |
|  | 60-69 | 1.00 | 1.05 (0.92-1.21) |
|  | 70-79 | 1.00 | 1.03 (0.95-1.12) |
|  | 80-85 | 1.00 | 1.02 (0.93-1.11) |
| **Sex** | Male | 1.00 | 1.02 (0.96-1.10) |
|  | Female | 1.00 | 1.03 (0.95-1.12) |
| **Tumor location** | Colon | 1.00 | 1.04 (0.98-1.11) |
|  | Rectum | 1.00 | 0.99 (0.90-1.09) |
| **Disease stage** | Stage I | 1.00 | 1.17 (1.03-1.43) |
|  | Stage II | 1.00 | 1.07 (0.98-1.17) |
|  | Stage III | 1.00 | 0.96 (0.89-1.04) |

** Estimated from Cox regression interaction models adjusted for sex, year of diagnosis, Charlson comorbidity index, hypertension, and ASA score.

**S TABLE 3** Hazard ratios (HRs) with 95% confidence intervals (CIs) comparing all-cause (top) and CRC-specific (bottom) mortality between aspirin users/non-users at CRC surgery, with no restricted follow-up time.

| **All-cause mortality** | **HR^a^ (95% CI)** | **HR^b^ (95% CI)** | **HR^c^ (95% CI)** |
| --- | --- | --- | --- |
| Non-aspirin use | 1.00 (ref) | 1.00 (ref) | 1.00 (ref) |
| Aspirin use* | 1.65 (1.57-1.73) | 1.24 (1.18-1.30) | 1.02 (0.97-1.08) |
|  |  |  |  |
| **CRC-specific mortality** |  |  |  |
| Non-aspirin use | 1.00 (ref) | 1.00 (ref) | 1.00 (ref) |
| Aspirin use* | 1.27 (1.78-1.36) | 1.07 (0.99-1.15) | 0.98 (0.91-1.06) |

**Defined as having at least one prescription of aspirin equaling to or exceeding a total of 180 days dispense, within the year prior to CRC-surgery date/index date.*

*^a^* Estimated from an unadjusted Cox regression model.

^b^ Estimated from a model as in (a) also adjusting for age at diagnosis, sex, and year of diagnosis.

^c^ Estimated from a model as in (b) also adjusting for Charlson comorbidity index, hypertension, and ASA score.

**S TABLE 4** Frequencies and proportions of baseline characteristics among the 193,165 comparators (matched 1:6 on age, sex, and year of diagnosis to CRC patients) stratified by aspirin exposure at index date

|  |  | **Aspirin user** | **Non-user** | **Total** | **HR** (95% CI)** |
| --- | --- | --- | --- | --- | --- |
| **Overall**, n (row%) |  | 41,083 (21.3) | 152,082 (78.7) | 193,165 (100) | - |
| **Dead***, n (col%) |  | 10,571 (25.7) | 17,929 (11.8) | 28,500 (14.8) |  |
| **Year of diagnosis**  n (col%) | 2007-2011 | 19,874 (48.4) | 69,662 (45.8) | 89,205 (46.3) | 1.00 |
|  | 2012-2016 | 21,209 (51.6) | 82,420 (54.2) | 103,629 (53.7) | 0.81 (0.79-0.83) |
| **Age at diagnosis**  n (col%) | 18-49 | 66 (0.2) | 9,551 (6.3) | 9,617 (5.00) | 0.11 (0.09-0.14) |
|  | 50-59 | 1,062 (2.6) | 19,703 (13.0) | 20,765 (10.8) | 0.38 (0.35-0.42) |
|  | 60-69 | 7,870 (19.2) | 46,934 (30.9) | 54,804 (28.4) | 1.00 |
|  | 70-79 | 19,732 (48.0) | 54,084 (35.6) | 73,816 (38.2) | 2.78 (2.68-2.89) |
|  | 80-85 | 12,353 (30.1) | 21,810 (14.3) | 34,163 (17.7) | 6.93 (6.67-7.19) |
|  | Mean age (sd) | 74.8 (7.0) | 68.1 (10.8) |  | - |
| **Sex** | Male | 24,939 (60.7) | 78,470 (51.6) | 103,409 (53.5) | 1.00 |
| n (col%) | Female | 16,144 (39.3) | 73,612 (48.4) | 89,756 (46.5) | 0.80 (0.78-0.82) |
| **Charlson comorbidity index** **(CCI)**  n (col%) | 0 | 17,632 (42.9) | 111,165 (73.1) | 128,797 (67.7) | 1.00 |
|  | 1 | 8,530 (20.8) | 12,287 (8.1) | 20,817 (10.8) | 2.72 (2.63-2.81) |
|  | ≥2 | 14,921 (36.3) | 28,630 (18,8) | 43,551 (22,6) | 3.8 (3.72-3.92) |

Abbreviations: HR; hazard ratio, CI; confidence interval, n; number, col; column

* Restricted to the first 6 years after diagnosis.

** Estimated from univariable Cox regression models.

**S FIGURE 1** Overall survival (OS) proportions estimated with the Kaplan-Meier method (left panel) and standardised over age, sex, year of diagnosis, Charlson comorbidity index and hypertension (right panel), by aspirin exposure at index date among colorectal cancer patients and matched comparators.
